# Mosquito-disseminated diflubenzuron and spinosad as alternatives to mosquito-disseminated pyriproxyfen: a proof-of-concept, blind, controlled comparison in experimental cages

**DOI:** 10.1590/0074-02760250160

**Published:** 2025-12-15

**Authors:** Gabriela Brandina Aquino de Abreu, Ayrton Sena Gouveia, Francisco Augusto da Silva Ferreira, José Joaquín Carvajal-Cortés, Cláudia Torres Codeço, Fernando Abad-Franch, Sérgio Luiz Bessa Luz

**Affiliations:** 1 Fundação Oswaldo Cruz-Fiocruz, Instituto Leônidas e Maria Deane, Núcleo de Patógenos, Reservatórios e Vetores na Amazônia, Manaus, AM, Brasil; 2 Fundação Oswaldo Cruz-Fiocruz, Instituto Oswaldo Cruz, Programa de Pós-Graduação em Biologia Parasitária, Rio de Janeiro, RJ, Brasil; 3 Fundação Oswaldo Cruz-Fiocruz, Instituto Oswaldo Cruz, Programa de Computação Científica, Rio de Janeiro, RJ, Brasil; 4 Universidade Federal de Minas Gerais, Instituto de Ciências Biológicas, Departamento de Parasitologia, Laboratório de Ecologia de Parasitos e Vetores, Belo Horizonte, MG, Brasil

**Keywords:** mosquito control, Aedes, insecticide auto-dissemination, resistance management

## Abstract

**BACKGROUND:**

Mosquito-disseminated pyriproxyfen (MD-PPF) is a promising novel tool for urban-mosquito control, yet resistance to PPF (a juvenile-hormone analogue) may arise in exposed mosquito populations. Alternative larvicide/pupicide molecules suitable for mosquito-driven dissemination, but with distinct modes of action, are therefore needed.

**OBJECTIVES:**

To provide a proof-of-concept evaluation of mosquito-disseminated diflubenzuron (MD-DFB, a chitin-synthesis inhibitor) and spinosad (MD-SPN, a biological neurotoxin composite) as potential alternatives to MD-PPF.

**METHODS:**

We studied *Aedes aegypti*-driven dissemination in 20 blind, controlled experiments run in 110 × 90 × 30-cm cages. Of primary interest was whether and how (a) mosquito-driven dissemination affected adult-mosquito emergence (1705 larvae in 40 open and 20 closed cups set inside cages; generalised linear mixed models) and (b) exposure to larvicide/pupicide-treated dissemination stations affected adult-female lifespan (400 females released inside cages; proportional-hazards mixed models).

**FINDINGS:**

Adult-mosquito emergence was similar across treatments in closed cups. In open cups, average emergence fell from ~90% [95% confidence interval (CI), 84-95%] in control cages to ~30% (20-43%), ~56% (42-69%), and ~75% (63-85%) in, respectively, MD-PPF, MD-DFB, and MD-SPN cages. Exposure to SPN, but not to DFB or PPF, clearly reduced adult-female lifespan (SPN death-hazard ratio 2.4; 1.2-5.0).

**CONCLUSION:**

Mosquito-disseminated diflubenzuron holds promise as a potential alternative to MD-PPF; further testing in field settings seems warranted.

Mosquitoes are notorious biting pests and the vectors of many human pathogens including viruses (dengue, Zika, chikungunya, yellow fever and more) and filarial worms.^1^ ^,^ ^2^ ^,^ ^3^ ^,^ ^4^ Mosquito species adapted to urban habitats are particularly dangerous, because they can fuel pathogen spread across large human populations.^5^ ^,^ ^6^ ^,^ ^7^ Urban-mosquito control is, therefore, critical to protecting public health, yet none of the many tactics tested to date has been fully successful.^1^ ^-^ ^7^ One key drawback is that the aquatic habitats in which urban mosquito juveniles (most notably *Aedes aegypti* juveniles) develop are often small, cryptic, and hidden or inaccessible, and therefore remain untreated or unmanaged during vector-control activities; as a result, the fraction of mosquito juvenile aquatic habitats that are effectively treated or managed (*i.e.*, ‘breeding-site coverage’) is usually low, and this may reduce the effectiveness of mosquito control interventions.^8^ ^,^ ^9^ ^,^ ^10^

One promising novel strategy involves using the mosquitoes themselves to disseminate pyriproxyfen (PPF), a potent insect juvenile-hormone analogue, from lure ‘dissemination stations’ treated with fine PPF powder to otherwise untreated aquatic larval habitats.^11^ ^,^ ^12^ ^,^ ^13^ ^,^ ^14^ This strategy, known as ‘PPF autodissemination’ or ‘mosquito-disseminated PPF’ (MD-PPF), has been shown in field trials to yield high breeding-site coverage (likely because urban *Aedes* spp. spread their eggs across several sites and prefer artificial containers),^13^ ^,^ ^14^ ^,^ ^15^ leading to sharp increases of juvenile-mosquito mortality and sharp declines of adult-mosquito emergence ― which, in turn, can lead to lower adult-mosquito densities and reduced dengue incidence.^15^ ^,^ ^16^ ^,^ ^17^ ^,^ ^18^ While these and other results^19^ suggest that MD-PPF can become a useful addition to the urban-mosquito control toolbox, experience shows that the large-scale, long-term use of insecticides has the potential to, and most likely will, select for resistant mosquitoes.^20^ ^,^ ^21^ ^,^ ^22^ ^,^ ^23^

Insecticide-resistance management depends on the availability of active ingredients with diverse modes of action and that can be deployed safely and conveniently at scale.^22^ In the case of mosquito-disseminated insecticides for urban-mosquito control, this translates into the need for chemicals that (i) effectively kill juvenile mosquitoes at the low doses that adult mosquitoes can pick and disseminate; (ii) do not knock-down or quickly kill adult mosquitoes (which would hamper dissemination); (iii) are formulated as powder particles suitable for mosquito-driven dissemination; (iv) have mechanisms of action that differ from that of the insecticide towards which mosquitoes have evolved resistance; and (v) are safe for humans and other vertebrates at the low concentrations expected to derive from mosquito-driven dissemination to mosquito larval habitats ― which, in practice, means having been evaluated and approved for use in drinking water by the World Health Organization (WHO).^24^ ^,^ ^25^ ^,^ ^26^ ^,^ ^27^

We conducted a proof-of-concept study to test whether a chitin-synthesis inhibitor (diflubenzuron, DFB) and a biological neurotoxin composite (spinosad, SPN), both WHO-approved and in regular use for mosquito control,^26^ ^,^ ^27^ may be useful for urban-mosquito control strategies based on mosquito-driven dissemination. Using replicate blind, controlled experiments, we evaluated (i) whether *Ae. aegypti* females disseminate PPF, DFB, and SPN from dissemination stations (DSs) to untreated larval habitats inside experimental cages; (ii) the effects of such dissemination on juvenile *Ae. aegypti* development; and (iii) whether and how exposure to insecticide-treated DSs would affect the lifespan and death hazard of adult *Ae. aegypti* females. While we expected SPN to shorten adult-female lifespan, we wanted to test whether, in the specific context of mosquito-driven dissemination, this effect was slow enough to allow for some measurable degree of larvicide spread from DSs. As it turned out, SPN quickly killed many females, and only DFB emerged as a potentially useful alternative or complement to pyriproxyfen for mosquito-driven dissemination.

## MATERIALS AND METHODS

*Mosquitoes* - Using oviposition traps, we collected *Ae. aegypti* eggs from two neighbourhoods of Manaus, Amazonas, Brazil and established a laboratory colony; we then used first-generation offspring from that colony in all our experiments.

*Insecticides* - We used commercially available formulations of PPF (0.5% granules; Sumitomo Chemical, Tokyo, Japan), DFB (25% wettable powder; Champion Farmoquímico Ltd., Anápolis, Brazil), and SPN (7.48% effervescent tablets; Clarke Mosquito Control Inc., Roselle, USA). PPF granules and SPN tablets were ground to fine, talcum-like powder for use in the experimental procedures detailed below.

*Controls* - We treated PPF, which has been tested extensively for mosquito-driven dissemination,^11^ ^-^ ^19^ as our positive control in all experiments. As a negative control (abbreviated ‘CTR’ hereafter), all experiments included replicates using a sham ‘treatment’ or ‘placebo’ with a non-insecticidal powder ― fine pumice powder (Asfer, São Caetano do Sul, Brazil), which is similar in appearance to the insecticide powders we used.

*Blinding* - Experimenters evaluating treatment effects on any outcome (juvenile development and mortality, adult-female lifespan and death hazard) were blinded to the identity of the products used in each replicate ― whether PPF, DFB, SPN, or CTR. For this, research team members not involved in outcome evaluation labelled all items (bottles, flasks, DSs, cups, cages, etc. as detailed below) with unique codes to which experimenters involved in outcome evaluation did not have access. Codes were linked back to treatments only after all experiments were completed.

*Diagnostic-concentration bioassays* - We first did standard diagnostic-concentration larval bioassays^28^ to test whether our mosquito population was susceptible to PPF, DFB, and SPN. Diagnostic concentrations were defined as approximately twice the 99% lethal concentration (LC_99_) of active ingredient;^28^ based on published results, we used 0.02 parts per million (ppm) for PPF, 1.5 ppm for DFB, and 1 ppm for SPN.^29^ ^,^ ^30^ ^,^ ^31^ We prepared stock solutions by mixing (over 1 h on a shaker) 0.5 L of distilled water with either PPF (5 g of 0.5% powder = 50 ppm); DFB (5 g of 25% powder = 2500 ppm); SPN (6.7 g of 7.48% powder = 1000 ppm); or pumice powder (5 g). Each stock solution was stored in a glass bottle wrapped with aluminium foil and coded with a randomly assigned letter (A, B, C or D). We hatched 800 *Ae. aegypti* eggs from our colony and reared the larvae to stage LIII; we then separated 40 cohorts of 20 larvae each in 200-mL individually-coded plastic cups with 100 mL of tap water and a pinch of fish food. A researcher blinded to stock solution identity then pipetted, using disposable tips, a prescribed amount of each stock solution ― 40 μL for stock solution B (containing PPF), 60 μL for stock solution D (with DFB), 100 μL for stock solution A (SPN), and 50 μL for stock solution C (pumice powder) ― into each individually-identified cup, and gently stirred the mix using a disposable stick. The cups were then closed with cotton cloth and checked daily (by blinded evaluators) to record dead larvae or pupae (which were removed using disposable plastic pipettes) and emerging adults (removed using a Castro aspirator) until all 20 juveniles in each cup had either died or emerged as adults.

**Fig. 1:**
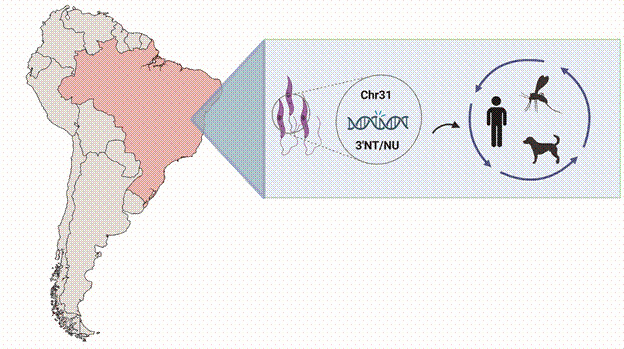
cages used in mosquito-driven dissemination experiments. Cages (110 × 90 × 30 cm) were built using metal frame, PVC panels, and nylon mesh. (A) frontal view, showing a dissemination station placed on a small raised shelf; (B) lateral view, showing also the three small oviposition cups placed on the bottom of the cage.

**Fig. 2:**
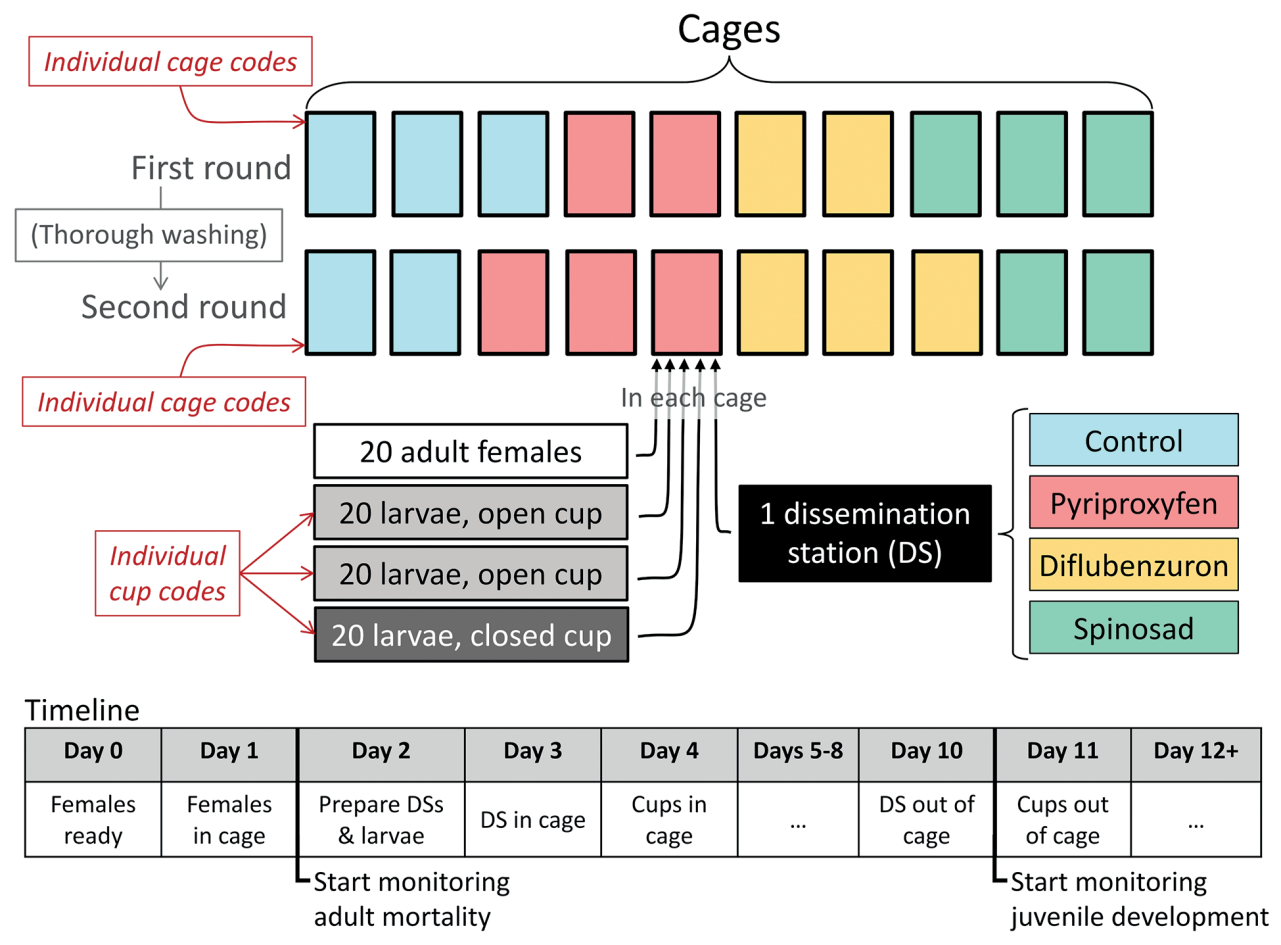
design of mosquito-driven dissemination cage experiments. Ten cages were used in two rounds; each cage had one insecticide dissemination station (four treatments: pyriproxyfen, diflubenzuron, spinosad, and pumice powder), three oviposition cups (two open and one closed) with 20 *Aedes aegypti* larvae each, and 20 *Ae. aegypti* adult females. Codes were used to keep researchers evaluating mosquito outcomes (juvenile mortality, adult lifespan and death hazard) blind to treatment. The lower panel shows the experiment timeline for each of the two rounds.

*Mosquito-driven dissemination: cage experiments* - We built ten 110 × 90 × 30-cm mosquito cages (Fig. 1) to run the dissemination experiments; we ran two replication rounds, with cages thoroughly washed between rounds (Fig. 2). We used digital thermo-hygrometers (1566-1, J.Prolab, São José dos Pinhais, Brazil) and thermometers (TDU-100, Unity, Guarulhos, Brazil) to record daily mean, maximum, and minimum values of (i) temperature (ºC) in each cage and (ii) relative humidity (RH, %) in two rooms, each housing five cages per round. Within each cage, we placed one DS on a small raised shelf and three oviposition cups on the cage floor (Figs. 1-2); 20 mated and blood-fed, 7-10 days-old *Ae. aegypti* females were then released inside each cage and supplied with water and 10% sucrose solution in separate cotton balls, which were replaced twice weekly. DSs were 2-L plastic cups (Fig. 1) filled with ~1800 mL of tap water and with the walls lined with black cloth dusted with ~5 g/m^2^ of either PPF, DFDB, SPN, or CTR powder.^13^ ^,^ ^15^ Each oviposition cup had 100 mL of tap water with fish food, a strip of filter paper that mosquito females could use for egg laying, and 20 *Ae. aegypti* LIII larvae hatched from our colony (see above). Two of the cups were open, and hence available for contamination with mosquito-disseminated insecticide, whereas the third cup was closed with nonwoven fabric (from disposable lab coats) and was meant to act as an ‘in-cage’ negative control. We removed DSs and oviposition cups from the cages after seven days. Adult mosquitoes remained inside the cages until death or for up to 60 days.

To measure the possible effects of mosquito-driven dissemination on juvenile-mosquito development, blinded evaluators checked the cups daily as in the bioassays described above. To measure the possible effects of exposure to insecticide powder on adult female lifespan and death hazard, blinded evaluators checked the cages daily for dead specimens.

*Data analysis* - We first describe and summarise our data in tables and graphs; for proportions, we compute score 95% confidence intervals (CI) using *Hmisc* 5.2-3.^32^ We then use mixed models including (i) binomial generalised linear mixed models (logit link-function), fitted using *glmmTMB* 1.1.10,^33^ for assessing treatment effects on adult-mosquito emergence; and (ii) proportional-hazards (Cox) mixed models, fitted using *coxme* 2.2-22,^34^ for assessing treatment effects on adult-mosquito lifespan. These models (a) incorporate fixed effects of the four-level treatment factor (and, in the case of cage experiments, an interaction between treatment and open cup), (b) adjust for covariates where relevant, and (c) explicitly account for the non-independence of observations stemming from the same cup and the same cage, as appropriate, via random intercepts. When more than a single model was fitted to the same data, we used Schwartz’s information criterion (also known as the Bayesian information criterion, or BIC)^35^ to assess relative model performance, and then focused on the results from the best-performing model; we used *ggeffects* 2.1.0^36^ to compute marginal mean predictions and CIs from those focal models. Details on model structure and specification are given in the RESULTS section to avoid repetition. All these analyses were run on an intention-to-treat basis (*i.e.*, ignoring possible treatment leakage into control, closed cups; see RESULTS) in R v 4.4.2.^37^ The datasets and R code needed to reproduce the results of this report are available at Figshare (https://doi.org/10.6084/m9.figshare.30265336.v1).

## RESULTS

**Fig. 3:**
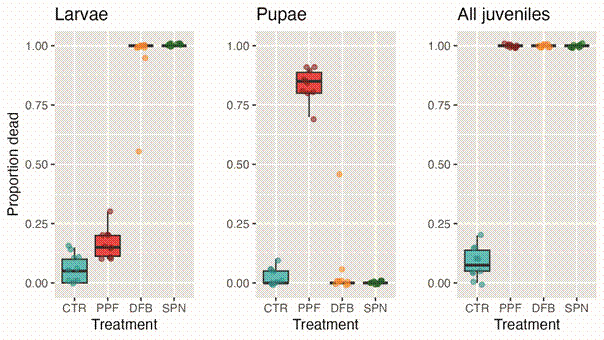
diagnostic-concentration bioassay results. *Aedes aegypti* juvenile mortality (given as the proportion of larvae, pupae, and larvae + pupae that died before reaching adulthood) caused by diagnostic concentrations of pyriproxyfen (PPF), diflubenzuron (DFB), and spinosad (SPN); a negative control (CTR, pumice powder) was also tested. Circles give mortality in each of 10 replicates (20 larvae each) per treatment. Boxplots highlight median values (thick horizontal line) and inter-quartile ranges (IQR; boxes); upper whiskers extend from the upper quartile (Q3) to either Q3 + 1.5 × IQR or the maximum observed value (whichever is smaller), and lower whiskers from the lower quartile (Q1) to either Q1 - 1.5 × IQR or the minimum observed value (whichever is larger).

*Diagnostic-concentration bioassays* - Juvenile mortality was 100% in all insecticide treatments, *vs*. ~9% in the control group; as expected after the mode of action of each insecticide, DFB and SPN killed mainly larvae, whereas PPF killed mainly pupae (Fig. 3). These results confirmed that the local *Ae. aegypti* population used in our experiments was susceptible to PPF, DFB, and SPN ― and that our choice of pumice powder as a ‘placebo’ negative control was well justified.

**Fig. 4:**
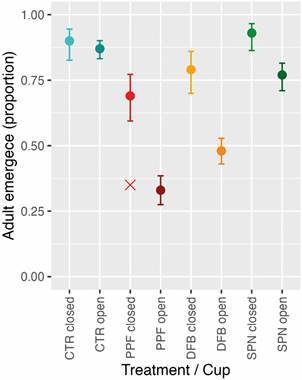
observed effects of mosquito-disseminated pyriproxyfen (PPF), diflubenzuron (DFB), and spinosad (SPN) on *Aedes aegypti* adult emergence: results from cage experiments including a negative control group (CTR). For each treatment, we show the average (circle) and the score 95% confidence interval (CI; error bars) both for closed (lighter shade; ‘in-cage’ negative controls, five per treatment) and open oviposition cups (darker shade; 10 per treatment). The red ‘×’ symbol highlights low adult-emergence from an ‘in-cage’ negative control (closed) cup that likely became contaminated with PPF; if we exclude this point from the calculations, average adult emergence from closed cups inside PPF cages rises to 0.78 (95% CI, 0.67-0.85) ― almost the same as for DFB (0.79; 95%CI, 0.70-0.86).

*Mosquito-driven dissemination: cage experiments* - We monitored juvenile development in a total of 1705 *Ae. aegypti* ― the 1200 larvae we placed inside oviposition cups within the experimental cages plus 505 larvae hatched from eggs laid by the mated females we released in the cages. Table I presents a summary of our observations, stratified by treatment and oviposition cup type (open or closed). Relative to the control group, there was a sharp decline in observed adult emergence from open cups in the PPF and DFB treatments, but only a modest decline in the SPN treatment. As expected, PPF induced mortality preferentially at the pupal stage, whereas DFB and (to a lesser extent) SPN induced mortality preferentially at the larval stage (Table I). Adult emergence from closed cups was also visibly reduced in PPF cages and perhaps slightly reduced in DFB cages, suggesting that some closed cups might have become contaminated. In particular, only seven of the 20 larvae (35.0%) in one closed cup set within a PPF cage completed development and emerged as adults ― a value that is apparently compatible with those seen among open cups in the same treatment (32.7% on average; score 95% CI, 27.5-38.5%; range, 12.5-50.0%) (Table I, Fig. 4).

**TABLE I:** Effects of mosquito-disseminated pyriproxyfen (PPF), diflubenzuron (DFB), and spinosad (SPN) on *Aedes aegypti* juvenile (larval and pupal) mortality and adult emergence: observed results, given as number (proportion of total), from cage experiments including a negative control group (CTR)

Treatment	Cup^*^	Total juveniles	Dead as larvae	Dead as pupae	Emerging as adults
CTR	Open	362	42 (0.116)	5 (0.014)	315 (0.870)
	Closed	100	4 (0.040)	6 (0.060)	90 (0.900)
PPF	Open	278	50 (0.180)	137 (0.493)	91 (0.327)
	Closed	100	19 (0.190)	12 (0.120)	69 (0.690)
DFB	Open	407	171 (0.420)	41 (0.101)	195 (0.479)
	Closed	100	10 (0.100)	11 (0.110)	79 (0.790)
SPN	Open	258	37 (0.143)	23 (0.089)	198 (0.767)
	Closed	100	7 (0.070)	0 (0.000)	93 (0.930)

*oviposition cups placed inside cages; ‘open’ cups (10 per treatment) were open and hence available for contamination by egg-laying females, whereas ‘closed’ cups (five per treatment) were closed with nonwoven fabric (from disposable lab coats) and acted as ‘in-cage’ negative controls.

**Fig. 5:**
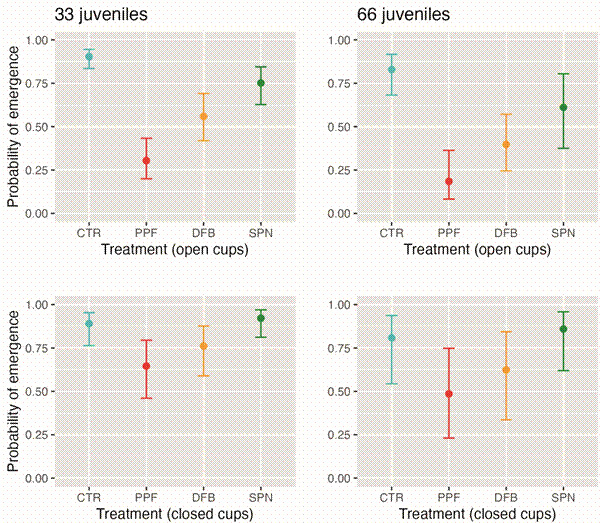
effects of mosquito-disseminated pyriproxyfen (PPF), diflubenzuron (DFB), and spinosad (SPN), compared to a negative control (pumice powder; CTR), on *Aedes aegypti* adult emergence from open (upper row) and closed oviposition cups (lower row): predictions from a binomial generalised linear mixed model. Circles show predicted probabilities of adult-mosquito emergence [error bars, 95% confidence intervals (CI) ]; to illustrate the small additive, negative effect of crowding on juvenile survival, we present predictions at the average observed juvenile-mosquito density in open cups during our experiments (33 juveniles per 100 mL of water) and twice that value.

To quantify treatment effects on juvenile-mosquito development, we ran binomial generalised linear mixed models (GLMMs) with adult emergence (a binary indicator for each of the *N* = 1705 juvenile mosquitoes) as a function of treatment (four-level factor), cup (binary), and their interaction, and with random intercepts for individual cage (a 20-level factor) and cup (a 60-level factor) to account for cage- and cup-level dependencies. Using BIC scores, we identified juvenile-mosquito crowding (the scaled number of juveniles developing in each cup) as an important covariate; in contrast, differences between the two experimental rounds, as well as temperature and RH effects, were not significant. The numerical output of our focal, smallest-BIC model, which includes crowding as a covariate, is presented in Table II, and model-predicted probabilities of adult-mosquito emergence from open and closed cups across treatments and for two levels of juvenile-mosquito crowding are presented in Fig. 5. These results show that the effectiveness of mosquito-driven dissemination declined from PPF to DFB to SPN. They also suggest that there may have been some contamination of closed cups in cages with PPF, and perhaps also in cages with DFB; thus, our results likely underestimate to some extent the effectiveness of mosquito-driven dissemination for those two insecticides. There was statistical evidence of mosquito-driven dissemination of SPN, but the estimated impact on adult-mosquito emergence was small. Finally, there was an also small, negative effect (which our model adjusts for) of juvenile-mosquito crowding on juvenile survival to adulthood (Table II, Fig. 5).

**TABLE II:** Effects of mosquito-disseminated pyriproxyfen (PPF), diflubenzuron (DFB), and spinosad (SPN) on *Aedes aegypti* adult emergence: numerical output of a generalised linear mixed model

Term	Estimate	SE	*z*	p	CI lower	CI upper
Fixed effects						
Intercept	2.189	0.461	4.75	-	1.286	3.091
PPF^*a*^	-1.498	0.580	-2.53	0.0098	-2.636	-0.361
DFB	-0.937	0.593	-1.58	0.1138	-2.098	0.224
SPN	0.369	0.670	0.55	0.5821	-0.944	1.681
Open cup	0.138	0.494	0.28	0.7798	-0.831	1.107
PPF × open cup	-1.565	0.592	-2.65	0.0082	-2.725	-0.405
DFB × open cup	-1.059	0.594	-1.78	0.0743	-2.223	0.104
SPN × open cup	-1.496	0.687	-2.18	0.0295	-2.842	-0.149
Crowding (scaled)^*b*^	-0.228	0.114	-1.99	0.0465	-0.451	-0.004
Random intercepts (SD)						
Cup (*n* = 60)	0.442	-	-	-	0.275	0.711
Cage (*n* = 20)	0.459	-	-	-	0.260	0.808

*a*: the clearly negative coefficient of this main term suggests that there was contamination of closed oviposition cups in cages with PPF-treated dissemination stations (see also Fig. 4); *b*: number of juveniles developing in each cup, scaled to mean 0 and SD 1 (observed mean = 28.42; observed SD = 11.44); SE: standard error; z: z-statistic; p: p-value associated with z; CI lower and CI upper: lower and upper limits of the 95% confidence interval; SD: standard deviation.

**Fig. 6:**
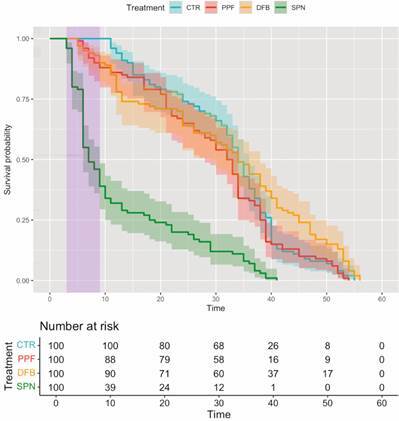
effects of exposure to dissemination stations (DSs) treated with pyriproxyfen (PPF), diflubenzuron (DFB), or spinosad (SPN), compared to a negative control (pumice powder; CTR), on the lifespan of caged *Aedes aegypti* adult females: Kaplan-Meier survival curves (coloured lines) and log-log 95% confidence intervals (coloured strips around lines) over up to 60 days (‘Time’, x-axis) of daily observations. The purple vertical band at days 3-9 highlights the time-period during which DSs remained inside the cages. The lower table shows the numbers of females that were still alive at seven time-points in each treatment.

We also aimed at measuring the effects of exposure to insecticide-treated DSs on the lifespan of *Ae. aegypti* adult females (*N* = 400). We first tabulated survival data and plotted Kaplan-Meier survival curves to show that, relative to exposure to sham-treated DSs (CTR), exposure to SPN-treated DSs clearly shortened female lifespan, whereas exposure to PPF- or DFB-treated DSs did not (Fig. 6). Fig. 6 also shows that mortality rates in SPN cages were particularly high early in the experiments, and especially over the seven days when DSs were still within the cages (purple band in Fig. 6). This suggests that contact with SPN-treated DS surfaces was quickly lethal for many adult females.

**Fig. 7:**
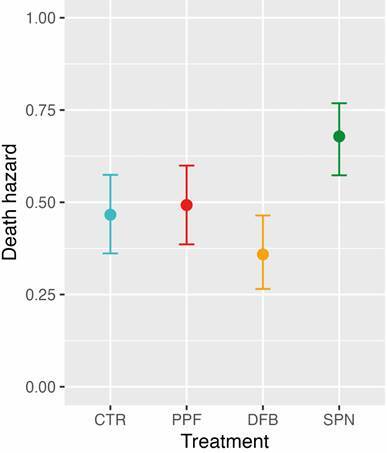
effects of exposure to dissemination stations treated with pyriproxyfen (PPF), diflubenzuron (DFB), or spinosad (SPN), compared to a negative control (pumice powder; CTR), on the death hazard of caged *Aedes aegypti* adult females: predictions from a Cox mixed-effects proportional-hazards model. Predictions are given on the probability scale; they correspond to expectations at average values of minimum temperature and maximum relative humidity, and take into account the non-independence of observations made in each of 20 experimental replicates (five per treatment).

To get adjusted estimates of instantaneous death-hazards across treatments, we ran a series of Cox proportional-hazards mixed-effects models with treatment fixed effects, cage-level random intercepts, and varying covariate structure. BIC-score comparisons selected a model including (scaled) minimum temperature (‘t_min’) and maximum RH (‘rh_max’) as covariates; Table III shows the numerical output of this model. The clearly positive effect of SPN corresponds to an adjusted death-hazard ratio of 2.42 (95% CI, 1.17-5.01), relative to the CTR group baseline hazard; Fig. 7 shows adjusted death hazards as predicted by the model at the observed average t_min (25.6ºC) and rh_max (88.2%).

**TABLE III:** Effects of exposure to dissemination stations treated with pyriproxyfen (PPF), diflubenzuron (DFB), or spinosad (SPN) on the lifespan of caged *Aedes aegypti* adult females: numerical output of a Cox proportional-hazards mixed-effects model

Term	Estimate	SE	*z*	p	CI lower	CI upper
Fixed effects						
PPF	0.105	0.361	0.29	0.7733	-0.602	0.811
DFB	-0.447	0.360	-1.24	0.2149	-1.152	0.259
SPN	0.882	0.372	2.37	0.0177	0.153	1.611
t_min (ºC, scaled)	1.504	0.112	13.39	< 0.0001	1.284	1.724
rh_max (%, scaled)	1.184	0.118	10.02	< 0.0001	0.952	1.415
Random intercepts (SD)^*^						
Cage (*n* = 20)	0.519	-	-	-	0.463	0.778

*coxme 2.2-22 does not provide CIs for random-effect estimates; the CI limits given here were computed using non-parametric bootstrapping (1000 replicates). SE: standard error; *z*: *z*-statistic; p: p-value associated with *z*; CI lower and CI upper: lower and upper limits of the 95% confidence interval; t_min, minimum daily temperature; rh_max, maximum daily relative humidity; SD: standard deviation.

## DISCUSSION

The large-scale deployment of insecticides to control disease vectors often leads to the selection of resistant vector populations; insecticide-resistance management is, therefore, a key component of rational vector control.^20^ ^-^ ^22^ ^,^ ^38^ ^-^ ^40^ As novel insecticide-based control strategies are developed, resistance management critically depends on the availability of active ingredients with diverse modes of action and favourable product profiles ― *i.e.*, suitably formulated, safe, and cost-effective products.^38^ ^,^ ^39^ ^,^ ^41^ One promising novel strategy for urban mosquito control is mosquito-driven dissemination of insecticides,^42^ yet just one active ingredient ― the juvenile-hormone analogue, PPF ― has so far been shown to be effective in field trials.^13^ ^-^ ^19^ Using replicate, blind, controlled cage experiments, here we tested two alternative mode-of-action products potentially suitable for mosquito-driven dissemination ― a chitin-synthesis inhibitor (DFB)^26^ and a biological neurotoxin composite (SPN).^27^ We found that *Ae. aegypti* females can disseminate DFB and SPN to untreated larval habitats, leading to reduced adult-mosquito emergence. Exposure to SPN, however, quickly killed many adult females ― an acute-toxicity effect that apparently hampered SPN dissemination. DFB thus emerges as a potentially useful alternative or complement to PPF for mosquito-driven dissemination in the context of insecticide-resistance management based on rotations or combinations.^22^ While DFB and PPF act on different biological processes and have different modes of action,^43^ their detoxification may involve some common biochemical pathways (*e.g.*, some cytochrome P450 enzymes);^44^ hence, resistance monitoring should consider not only resistance to PPF or DFB separately, but also the possibility of PPF/DFB cross-resistance ― which, to our knowledge, has so far not been identified in any *Aedes* field population.

We report a moderate reduction of adult-mosquito emergence from open oviposition cups set inside cages with one DFB-treated dissemination station (DS). At a density of ~33 larvae per 100 mL of water (the observed average in open cups), our focal adult-emergence model (Table II) predicts that just ~56.0% (95%CI, 41.9-69.1%) of *Ae. aegypti* juveniles will complete development, *vs*. a much higher 90.3% (95%CI, 83.5-94.5%) in control cages; by contrast, exposure to SPN-treated DSs reduced adult emergence more modestly (to a predicted average of 75.2%; 95%CI, 62.8-84.5%), and exposure to the much more potent PPF had the strongest effect (30.4% predicted emergence; 95%CI, 20.0-43.3% ― overall consistent with the results of previous field trials)^13^ ^,^ ^14^ ^,^ ^15^ ^,^ ^16^ (Fig. 5). Because we found some evidence suggesting leakage of PPF, and perhaps DFB (Figs. 4-5, Table II), into ‘in-cage’ negative controls (closed cups set inside test cages), our estimates of adult-emergence inhibition by these two compounds are likely underestimates. On the other hand, our model-based estimates adjust for the effects of larval crowding on juvenile development ― which, in line with prior studies,^45^ ^,^ ^46^ were negative: odds ratio = 0.797 (95%CI, 0.637-0.996) for each 1 SD (~11.4 larvae per 100 mL of water) increase in larval density (Table II, Fig. 5).

One important feature of candidate molecules for mosquito-driven dissemination is that they should not cause acute toxicity in adult mosquitoes ― if adults visiting a treated DS are quickly killed, knocked-out, or otherwise rendered unable to visit further larval habitats, then dissemination can be compromised. To see if exposure to treated DSs affected adult lifespan, we measured female-mosquito survival (*N* = 400 *Ae. aegypti* females) over 60 days in our experimental cages. We expected that exposure to SPN-treated DSs would shorten adult-female lifespan to some extent, yet perhaps slowly enough not to hinder dissemination. We found, however, that SPN quickly killed many adult females, with ~61% of them (*vs*. ~0-22% in the other treatments) dying in days 3-9 of the experiment ― *i.e.*, when DSs were still set inside the cages (Fig. 6). This translated into an adjusted SPN death-hazard ratio of ~2.42 (95%CI, 1.17-5.01; mixed-effects Cox model, Table III); overall female survival and death-hazards, in contrast, were similar in pumice-treated (control) cages and in PPF- and DFB-treated cages (Fig. 6). These results suggest that while SPN might be useful as a contact adulticide in lethal ovitraps,^47^ ^,^ ^48^ it is not a strong candidate for mosquito-driven dissemination ― a view that is further supported by the small reduction of adult emergence from open cups in SPN-treated cages (Fig. 5).

Our study has several limitations. First, while cage-experiment results provide crucial guidance and justification for field testing, their external validity needs to be established in real-world settings. Along the same lines, we did our tests on *Ae. aegypti* mosquitoes from Manaus because that is where we plan to run small-scale field trials, but whether and to what extent the results will replicate in mosquitoes with different genetic backgrounds remains to be seen. We also note that we did not experimentally define diagnostic concentrations for each test compound; instead, we retrieved those values from the literature.^29^ ^,^ ^30^ ^,^ ^31^ This was because, with that part of the study, we just aimed at making sure that (i) our mosquitoes were not resistant to the test active ingredients and (ii) those ingredients were indeed active and working as expected. Finally, we did not have the chemical-analytic means to measure active-ingredient concentrations in the water of oviposition cups set inside experimental cages; instead, we directly measured the entomological endpoints of interest (juvenile mortality and adult emergence) and assumed that any changes, relative to control cages and cups, were due to mosquito-driven dissemination. Determination of active-ingredient concentrations would also have helped us firmly establish whether there was leakage of PPF and DFB into closed cups inside treated cages ― and this, in turn, would have allowed us to measure the degree to which our results underestimate adult-emergence inhibition by those two compounds.

Taken together, in sum, our findings suggest that the chitin-synthesis inhibitor, DFB, but not the biological neurotoxin composite, SPN, may be useful for *Aedes* control in the context of mosquito-driven dissemination. These encouraging experimental results, together with prior theoretical work,^49^ indicate that small-scale field trials should proceed with the aim of testing whether deployment of DFB-treated DSs effectively leads to mosquito-driven dissemination in real-world settings ― and, if so, whether such dissemination yields suitable levels of larval-habitat coverage and leads to measurable reductions of adult-mosquito emergence and mosquito population densities. Other promising chitin-synthesis inhibitors, such as lufenuron^50^ and novaluron^51^ should also enter the mosquito-driven insecticide dissemination testing pipeline ― from laboratory bioassays and cage experiments such as those described here to, if the results are encouraging, field trials.

## Data Availability

The datasets and R code needed to reproduce the results of this report are available at Figshare (https://doi.org/10.6084/m9.figshare.30265336.v1).
